# Direct synthesis of extra-heavy olefins from carbon monoxide and water

**DOI:** 10.1038/s41467-023-37599-2

**Published:** 2023-04-03

**Authors:** Chuanhao Wang, Junjie Du, Lin Zeng, Zhongling Li, Yizhou Dai, Xu Li, Zijun Peng, Wenlong Wu, Hongliang Li, Jie Zeng

**Affiliations:** 1 Hefei National Research Center for Physical Sciences at the Microscale, Key Laboratory of Strongly-Coupled Quantum Matter Physics of Chinese Academy of Sciences, Key Laboratory of Surface and Interface Chemistry and Energy Catalysis of Anhui Higher Education Institutes, Department of Chemical Physics, University of Science and Technology of China, 230026 Hefei, Anhui P. R. China; 2 School of Chemistry & Chemical Engineering, Anhui University of Technology, 243002 Ma’anshan, Anhui P. R. China

**Keywords:** Heterogeneous catalysis, Catalyst synthesis, Chemical engineering

## Abstract

Extra-heavy olefins (C_12+_^=^), feedstocks to synthesize a wide range of value-added products, are conventionally generated from fossil resources *via* energy-intensive wax cracking or multi-step processes. Fischer-Tropsch synthesis with sustainably obtained syngas as feed-in provides a potential way to produce C_12+_^=^, though there is a trade-off between enhancing C-C coupling and suppressing further hydrogenation of olefins. Herein, we achieve selective production of C_12+_^=^ *via* the overall conversion of CO and water, denoted as Kölbel-Engelhardt synthesis (KES), in polyethylene glycol (PEG) over a mixture of Pt/Mo_2_N and Ru particles. KES provides a continuously high CO/H_2_ ratio, thermodynamically favoring chain propagation and olefin formation. PEG serves as a selective extraction agent to hinder hydrogenation of olefins. Under an optimal condition, the yield ratio of CO_2_ to hydrocarbons reaches the theoretical minimum, and the C_12+_^=^ yield reaches its maximum of 1.79 mmol with a selectivity (among hydrocarbons) of as high as 40.4%.

## Abstract (web summary)

Fischer-Tropsch synthesis is challenged by the trade-off between enhancing C-C coupling and suppressing further hydrogenation of olefins. Here the authors break this challenge by developing a universal strategy to synthesis extra-heavy olefins from carbon monoxide and water *via* the sustained release of hydrogen and selective extraction of olefins.

## Introduction

Extra-heavy olefins (C_12+_^=^), production of which are highly relative to fossil energy with energy-intensive processes, are extensively used as feedstocks to synthesize a wide range of value-added products such as synthetic lubricants, surfactants, sizing agents, drilling fluid, biodegradable detergents, new polymers, and plasticizers^1,2^. Conventionally, C_12+_^=^ are produced from energy-intensive wax cracking^3^ or multi-step Shell higher olefin process (SHOP) containing oligomerization, isomerization, and metathesis with ethylene as original feedstock^2^. The ever-increasing depletion of petroleum reserves is spurring the exploration of sustainable production routes from non-petroleum carbon resources. Fischer–Tropsch synthesis (FTS) offers an approach to produce hydrocarbons such as light olefins, aromatics, gasoline, and diesel directly from syngas which readily derives from coal, biomass, CO_2_, and natural gas (Eq. 1)^4–8^.

1 $${{{{{\rm{CO}}}}}}+{2{{{{{\rm{H}}}}}}}_{2}={{-{{{{{\rm{CH}}}}}}}_{2}-}+{{{{{{\rm{H}}}}}}}_{2}{{{{{\rm{O}}}}}}$$

However, under classic FTS conditions, there is a trade-off between enhancing C-C coupling for long-chain products (C_12+_) and suppressing further hydrogenation of olefins. For instance, though iron nanoparticles on α-alumina^8^, cobalt carbide nanoprisms^9^, and Oxide-Zeolite^10^ favored the formation of olefins, these catalysts exhibited limiting selectivity for C_12+_ products. For ruthenium (Ru)-based catalysts with intrinsically high activity for chain growth^11–13^, most of the produced long-chain hydrocarbons are paraffins instead of olefins^14^, because Ru is highly active for the hydrogenation of unsaturated hydrocarbons. Though efforts have been devoted to the production of C_12+_^=^ in a few case studies^15,16^, it is of a grand challenge to propose a universal strategy to massively synthesize C_12+_^=^.

To break this counterbalance, we propose an alternative reaction route, Kölbel–Engelhardt synthesis (KES), which is the coupling of the water gas shift (WGS) reaction (Eq. 2) and FTS (Eq. 1). This cascade reaction (Eq. 3) was proposed in last century to utilize the CO-rich syngas, though it was not widely recognized in the field of CO conversion, not to mention practically replacing the unit of WGS and FTS, considering excess CO_2_ production and inhibited chain propagation^17–19^. Xu et al. reported the feasibility of this process in a slurry reactor over Ru-based catalysts, and considerable chain propagation was achieved, though a large margin still existed, in terms of CO_2_ excess or inadequate C_5+_ production compared with conventional FTS^20^.

2 $${{{{{\rm{CO}}}}}}+{{{{{{\rm{H}}}}}}}_{2}{{{{{\rm{O}}}}}}={{{{{{\rm{CO}}}}}}}_{2}+{{{{{{\rm{H}}}}}}}_{2}$$

3 $$3{{{{{\rm{CO}}}}}}+{2{{{{{\rm{H}}}}}}}_{2}{{{{{\rm{O}}}}}}={2{{{{{\rm{CO}}}}}}}_{2}+{{-{{{{{\rm{CH}}}}}}}_{2}-}+{{{{{{\rm{H}}}}}}}_{2}{{{{{\rm{O}}}}}}$$

Herein, we achieved highly selective production of C_12+_^=^ *via* direct conversion of CO and stoichiometric water (KES) in the solvent of polyethylene glycol (PEG) over Ru-based catalysts. During the cascade reaction of WGS and FTS, the sustained release of hydrogen *via* WGS afforded continuously low hydrogen pressure which kinetically benefited both chain propagation and olefin formation during FTS. The phase-transfer agent, PEG, served as a selective extraction agent to increase the selectivity for olefins. A mixture of Pt/Mo_2_N and Ru particles, which served as active components for WGS and FTS, respectively, was applied as the catalyst. In PEG under 2 MPa of CO with a stoichiometric ratio (3:2) of CO:H_2_O at 200 °C for 10 h, the yield ratio of CO_2_ to hydrocarbons reached the theoretical minimum of 2 (Eq. 3), reflecting the perfect kinetic match of WGS and FTS. Moreover, the C_12+_^=^ yield reached its maximum of 1.79 mmol with a selectivity (among hydrocarbons) of as high as 40.4%. Universality was also studied by replacing the noble-meta-containing catalysts to noble-metal-free catalysts.

## Results

### Characterizations of KES catalysts

To validate the effectiveness of KES, we prepared a catalyst consisting of active components for WGS and FTS. Specifically, Pt/Mo_2_N was fabricated for WGS, since Mo-based materials have already been reported to dissociate water molecules at a relatively low temperature (~150 °C)^21–23^. Mo_2_N, instead of molybdenum carbides, was chosen to avoid carbon contamination. The mass loading of Pt was determined to be 2.0% according to inductively coupled plasma-atomic emission spectrometry (ICP-AES). This mass loading was reported active with the capability of stabilizing Mo-based surface by rapid consumption of oxygen species from water dissociation in WGS process^23^. Such a low mass loading accounted for the absence of diffraction peaks of Pt in the X-ray diffraction spectroscopy (XRD) patterns of Pt/Mo_2_N (Supplementary Fig. S1). The high-angle annular dark-field scanning transmission electron microscopy (HAADF-STEM) image of Pt/Mo_2_N showed that Pt particles with a main size of ~2.5 nm were densely deposited on the support (Supplementary Fig. S2). This dense distribution ensured the rapid reaction of oxygen species with CO* on Pt to avoid the oxidation of Mo_2_N^23^. For the production of C_12+_^=^, FTS catalyst was set to Ru particles due to its outstanding activity for the chain propagation^11–13^.

**Fig. 1:**
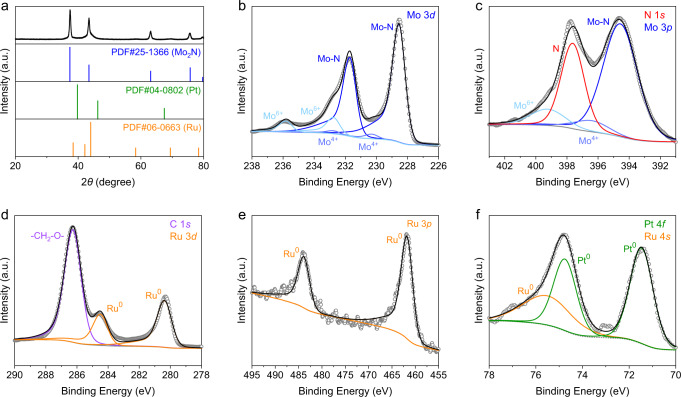
Structural characterizations of Pt/Mo_2_N-Ru. **a** XRD pattern of Pt/Mo_2_N-Ru. **b** Mo 3*d*, **c** N 1*s* and Mo 3*p*, **d** C 1*s* and Ru 3*d*, **e** Ru 3*p*, and **f** Pt 4*f* and Ru 4*s* XPS spectra of Pt/Mo_2_N-Ru.

The mixture of Pt/Mo_2_N and Ru particles, denoted as Pt/Mo_2_N-Ru, was used as the catalyst for KES. In a typical synthesis of Pt/Mo_2_N-Ru, RuCl_3_·*x*H_2_O, and Pt/Mo_2_N were dispersed in a reactor containing a mixture of PEG and water. Afterwards, the reactor was pressurized with hydrogen and heated to obtain Ru particles before the formal catalytic tests. As shown in the XRD patterns, a distinct set of peaks for Mo_2_N and a broad set of peaks for metallic Ru appeared (Fig. 1a). Figure 1b–f shows X-ray photoelectron spectroscopy (XPS) spectra of Pt/Mo_2_N-Ru. The presence of Mo^6+^ and Mo^4+^ was ascribed to inevitable surface oxidation during passivation in CO_2_ atmosphere (Fig. 1b, c)^24^. The C 1 *s* spectrum exhibited a peak at 286.3 eV which was assigned to the PEG residual on the surfaces of the catalyst (Fig. 1d). Based on Ru 3*d*, Ru 3*p*, Ru 4*s*, and Pt 4*f* spectra, both Ru and Pt were identified in metallic states (Fig. 1d–f). Notably, the Ru 3*d*_5/2_ peak located at 280.4 eV suggested negligible electronic interaction between PEG and Ru particles^25^. Supplementary Figure S3 shows scanning transmission electron microscopy-energy diffraction X-ray (STEM-EDX) elemental mapping images of Pt/Mo_2_N-Ru. We observed different distribution pattern of Ru from those of Mo, N, and Pt, suggesting that Ru particles were not supported on Pt/Mo_2_N. This was attributed to the capping effect of PEG, as reported elsewhere^26^. These small particles could have abundant step sites for CO activation^27^. For comparison, Ru deposited on Pt/Mo_2_N was also prepared, denoted as Ru/Pt/Mo_2_N, to verify the significance of the separation of Ru and Pt/Mo_2_N. EDX mapping of Ru/Pt/Mo_2_N indicated that Ru particles were mainly supported on Pt/Mo_2_N (Supplementary Fig. S4).

### Catalytic properties of Pt/Mo_2_N-Ru towards KES

**Fig. 2:**
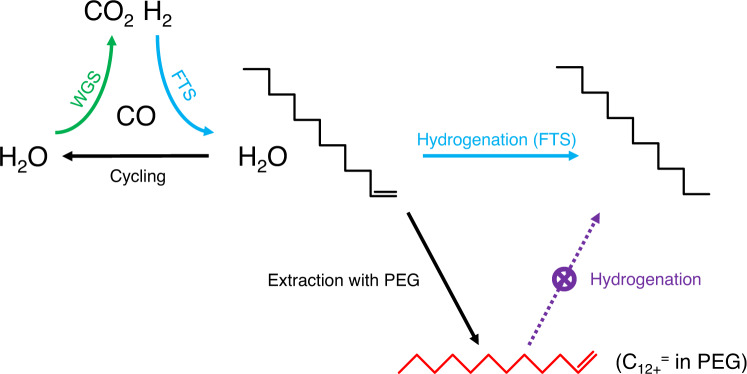
Scheme of sustained release of hydrogen *via* KES and selective extraction to promote the production of extra-heavy olefins. The green arrow represents WGS. The azure arrows represent FTS, containing chain propagation and hydrogenation. The dark violet arrow represents hydrogenation of dissolved olefins. For KES, hydrogen accumulates through WGS and is gradually consumed through FTS. Water by-produced from FTS serves again as reactant of WGS. Hence a relatively high CO/H_2_ ratio, which thermodynamically favors the production of C_12+_^=^, is maintained. Besides, a phase-transfer agent (PEG) with an affinity to olefins extracts olefin intermediates from surfaces of FTS catalysts to inhibit their further hydrogenation.

Despite hydrogen being invisible at both sides of Eq. (3), kinetically, hydrogen produced from WGS is necessary to drive FTS. Excess CO_2_ production reported before also suggested the difficulty to achieve this ideal condition. Though, different from a constant CO/H_2_ ratio in conventional FTS, in KES conducted in a slurry reactor hydrogen is sustainedly released from WGS and gradually consumed in FTS. This should provide a relatively high CO/H_2_ ratio, which favors chain propagation and suppresses the further hydrogenation of olefins. With this condition thermodynamically favorable for the production of C_12+_^=^, we still need the kinetic feasibility. Various solvents were applied when FTS was conducted in slurry reactors^28^. Phase-transfer agents exhibit affinity to both polar phases and non-polar phases^29^. PEG is one of the most widely used phase-transfer agents^30^. The characteristic affinity to organic compounds inspired us to explore the possibility of stabilizing olefins formed on the surfaces of catalysts. Hence, we first investigated the interaction between PEG and olefin. With cyclohexane as the extraction agent, 87% of 1-dodecene was extracted from PEG (PEG-400, unless mentioned specifically) (Supplementary Fig. S5). With the aid of a more polar solvent, water, this residue of 1-dodecene was completely expelled into cyclohexane solution, leading to an extraction efficiency of >99%. This contrast indicated a measurable interaction between PEG and olefins. As such, we assumed that PEG could potentially serve as an extractor for olefin intermediates formed during KES (Fig. 2).

**Fig. 3:**
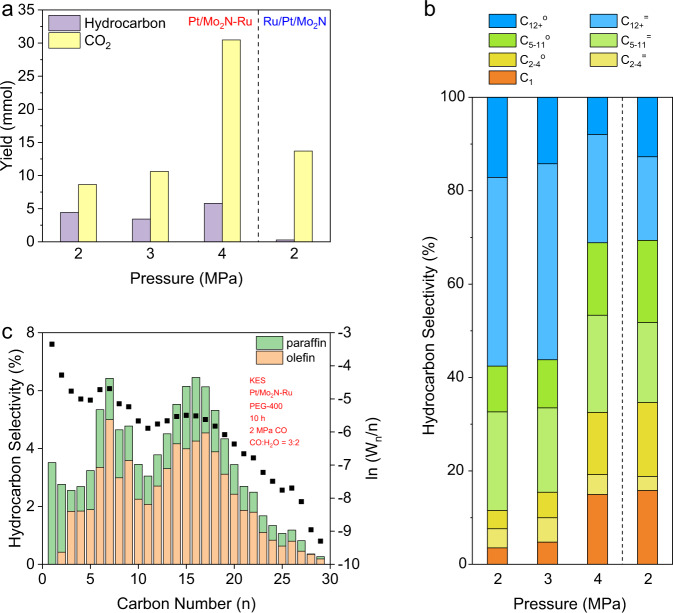
Catalytic performances of KES. **a** Yields of hydrocarbons and CO_2_ on a carbon-atom basis. From left to right: 2, 3, and 4 MPa of CO over Pt/Mo_2_N-Ru, and 2 MPa of CO over Ru/Pt/Mo_2_N. **b** Fractional hydrocarbon selectivity. From left to right: 2, 3, and 4 MPa of CO over Pt/Mo_2_N-Ru, and 2 MPa of CO over Ru/Pt/Mo_2_N. = represents olefins and o represents paraffins. **c** Detailed hydrocarbon selectivity over Pt/Mo_2_N-Ru under 2 MPa of CO. All catalytic experiments were conducted in a 50-mL Hastelloy slurry reactor at 200 °C for 10 h with a CO:H_2_O ratio of 3:2. Total volume of PEG solvent (with 284, 426, and 568 μL of water for 2, 3, and 4 MPa of CO) were kept to 15 mL. Pt/Mo_2_N-Ru contained 100 mg of Pt/Mo_2_N and ~37 mg of Ru particles, while 137 mg of Ru/Pt/Mo_2_N was used to ensure the same amounts of active components.

The catalytic tests were conducted over constant amounts of Pt/Mo_2_N-Ru in a mixture of PEG and water under different pressures of CO at 200 °C for 10 h, denoted as the standard condition. In this condition, the amount of water was tuned to keep the molar ratio of CO to H_2_O at 3/2 (Eq. 3), while the total volume of binary PEG/water solvent was kept to 15 mL. Notably, the oxygenate products were below the detection limit of gas chromatography (GC). When the pressure of CO increased from 2 MPa to 4 MPa, the yield of CO_2_ underwent a steady increase from 8.63 to 30.46 mmol under the standard condition (Fig. 3a, Supplementary Table S1), as expected from the volume contraction character of KES. In contrast, hydrocarbon production exhibited a saddle-shaped trend with the increasing pressure, along with the amount of water. Specially, the yield of hydrocarbons reached the minimum of 3.43 mmol under 3 MPa of CO, whereas the yields were 4.42 and 5.81 mmol under 2 and 4 MPa, respectively (Fig. 3a, Supplementary Table S1). As a result, the ratio of CO_2_ to hydrocarbons continuously raised with the elevated pressure of CO (Fig. 3a). It was worth noting that the ratio of 1.95 obtained under 2 MPa of CO was close to the stoichiometric CO_2_/–CH_2_– ratio of 2:1 as stated in Eq. (3). This result indicated the perfect match of WGS and FTS under specific condition. For hydrocarbon yield, the promotion effect of pressurization observed in conventional FTS process was absent here. The chain propagation was also inhibited at elevated pressures from the perspective of decreasing C_5+_ selectivity of 88.5% (2 MPa), 84.6% (3 MPa), and 67.4% (4 MPa) (Fig. 3b). This was quite different from FTS process at elevated pressures where CO* coverage was increased and thus more CH_x_* generated. We speculated this abnormal pressurization effect came from relatively sluggish kinetics of FTS, in consideration of all necessary hydrogen for FTS, a reaction of gaseous components, was produced *via* WGS, a reaction of a gaseous component and a dissolved component. CO diffusing into PEG phase were prone to directly react with OH* adsorbed on Pt/Mo_2_N instead of dissociating on the surface of Ru and waiting for diffusing hydrogen to form CH_*x*_.

It is worth noting that olefins took the dominance of hydrocarbon production, which contrasted both the major production of paraffins over undecorated Ru-based catalysts reported before^14^ and a higher temperature demanded for olefin production^4,10^. Detailed hydrocarbon distribution suggested chain propagation under 3 MPa of CO was similar to that under 2 MPa (Fig. 3c, Supplementary Fig. S6a), while 4 MPa of CO severely suppressed chain propagation (Supplementary Fig. S6b). We also observed that the ratio of olefins to paraffins among all C_2+_ products (o/p ratio) exhibited a positive relationship with the pressure of CO, whereas the selectivity for C_12+_ hydrocarbons took an opposite tendency (Fig. 3b). Although the selectivity for C_12+_^=^ among hydrocarbons under 2 MPa was 40.4%, slightly lower than that (41.9%) under 3 MPa, the C_12+_^=^ yield was optimized at 1.79 mmol under 2 MPa with other reaction parameters keeping the same as this standard condition (Fig. 3b, Supplementary Table S1). Thus, we denoted 2 MPa of CO along with a CO:H_2_O ratio of 3:2 as the optimal condition.

The matching degree of cascade reactions can also be adjusted by the intimacy of bifunctional components^31^. To this end, we explored the catalytic performance of Ru/Pt/Mo_2_N under the optimal condition (2 MPa of CO, CO:H_2_O = 3:2, 200 °C, 10 h). The CO_2_ yield increased to 13.70 mmol, while hydrocarbon production was severely suppressed with a yield of 0.27 mmol in comparison with the results over Pt/Mo_2_N-Ru (Fig. 3a, Supplementary Table S1). Considering that the majority of Ru was deposited on the surface of Mo_2_N, hydroxyl radicals generated on Mo_2_N *via* water dissociation reacted rapidly with CO adsorbed on Ru particles which behaved similarly to the supported Pt particles^21,23^. Hence, only a small fraction of CO* participated in the FTS process, resulting in a low yield of hydrocarbons. As a result of the mismatch of WGS and FTS, the hydrogen partial pressure over Ru/Pt/Mo_2_N became higher than that over Pt/Mo_2_N-Ru, disfavoring the formation of C_12+_^=^. As expected, the C_12+_^=^ selectivity (among hydrocarbons) of Ru/Pt/Mo_2_N was 17.9%, lower than that (40.4%) of Pt/Mo_2_N-Ru (Fig. 3b, Supplementary Fig. S7, Supplementary Table S1). Without regard to the detailed difference, the fractional hydrocarbon distribution over Ru/Pt/Mo_2_N under 2 MPa of CO was similar to that over Pt/Mo_2_N-Ru under 4 MPa of CO (Fig. 3b), suggesting the insufficiency of CO* over Ru particles under 4 MPa of CO.

### Mechanistic studies on KES to C_12+_^=^

**Fig. 4:**
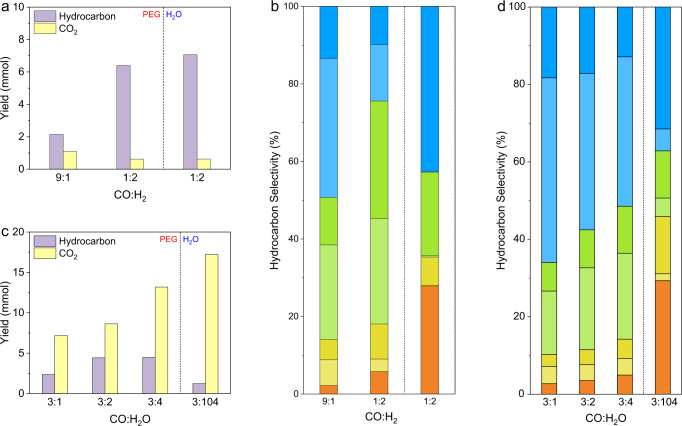
Mechanistic studies on KES. **a**, **b** FTS Catalytic performances for different ratios. **a** Yields of hydrocarbons and CO_2_ on a carbon-atom basis. From left to right: CO/H_2_ = 9:1 in PEG, CO/H_2_ = 1:2 in PEG, and CO/H_2_ = 1:2 in water. **b** Fractional hydrocarbon selectivity. From left to right: CO/H_2_ = 9:1 in PEG, CO/H_2_ = 1:2 in PEG, and CO/H_2_ = 1:2 in water. All catalytic experiments were conducted in a 50-mL Hastelloy slurry reactor over Ru particles at 200 °C under 2 MPa of syngas for 10 h. Volume of solvent (pure water or PEG) was kept to 15 mL. **c**, **d** Impact of water on KES catalytic performances. **c** Yields of hydrocarbons and CO_2_ on a carbon-atom basis. From left to right: molar ratios of CO to H_2_O of 3:1, 3:2, 3:4, and 3:104 (water as the solvent). **d** Fractional hydrocarbon selectivity. From left to right: molar ratios of CO to H_2_O of 3:1, 3:2, 3:4 (PEG-400 as the solvent), and 3:104 (water as the solvent). Colors in panels **b** and **d** represent the same products as those in Fig. 3b. All catalytic experiments were conducted in a 50-mL Hastelloy slurry reactor over Pt/Mo_2_N-Ru at 200 °C under 2 MPa of CO for 10 h. Total volume of solvent (pure water for CO:H_2_O ratio of 3:104 or PEG solvent with 142, 284, and 568 μL of water for CO:H_2_O ratio of 3:1, 3:2, and 3:4, respectively) were kept to 15 mL.

FTS experiments with syngas as feed-in were conducted to verify our assumption and speculation. Ru particles themselves with a main size of ~2.8 nm were fabricated with the same procedure for FTS tests (Supplementary Figs. S8 and S9). Under a conventional FTS gaseous condition (CO:H_2_ = 1:2) at 200 °C in different solvents (water and PEG), Ru particles exhibited different hydrocarbon selectivity in water and PEG (Fig. 4b, Supplementary Fig. S10, Supplementary Table S2), while the yields of CO_2_ and hydrocarbons were similar (Fig. 4a). Compared with the dominant production of paraffins in water, the use of PEG solvent did induce the formation of olefins with an o/p ratio of 0.92 (Supplementary Fig. S10), verifying our assumption that PEG could serve as an extractor and inhibit further hydrogenation of olefins. Difference of hydrocarbon distribution in PEG and water was also observed. Methane became the major product when water was used while a wide distribution was achieved in PEG (Supplementary Fig. S8c). We deduced that the polar character of water expelled CH_*x*_ intermediates on Ru surfaces, leading to a restricted chain distribution. When we turned the CO/H_2_ ratio from 1/2, a stoichiometric ratio for the FTS reaction (Eq. 1), to an extreme value of 9/1, the o/p ratio increased to 2.17 (Supplementary Fig. S8a, b, Supplementary Table S2), which was pretty close to that of KES under 2 MPa of CO (Supplementary Table S1). To investigate the possible CO-insertion mechanism for the chain growth, we conducted propylene hydroformylation over Ru particles in PEG. Unlike typical hydroformylation catalysts such as Rh, Ru particles exhibited an FTS-style behavior (Supplementary Fig. S11), while the oxygenates were still below the detection limit of GC. Therefore, the hydroformylation result, which fitted well with the absence of oxygenates in KES products, was not contradictory with the carbide mechanism which Ru-based catalysts preferred during FTS^32–34^. From FTS results, we concluded that maintaining a relatively high CO/H_2_ ratio with PEG as the solvent could induce the formation of olefins.

WGS directly affected the CO/H_2_ ratio, compared with hydrogen-consuming FTS which underwent sluggish diffusion of gaseous hydrogen to surfaces of Ru and activation on Ru. Hence, we should study the influence of water to the production of C_12+_^=^. In aforementioned KES experiments, limiting amount of water, though tightly coupled with CO pressure, played a vital role in the yield of C_12+_^=^ (Supplementary Table S1). We conducted catalytic tests over Pt/Mo_2_N-Ru with pure water and different amounts of water in PEG solvent under 2 MPa of CO at 200 °C for 10 h. When the solvent was changed from PEG to pure water, an extreme CO:H_2_O ratio of 3:104 was obtained. As expected, the yield of CO_2_ grew as the amount of water increased (Fig. 4c). The yield of hydrocarbons exhibited a volcano-type trend where the yield rose from 2.36 mmol (CO:H_2_O = 3:1) to 4.42 mmol (CO:H_2_O = 3:2) and 4.46 mmol (CO:H_2_O = 3:4) but finally descended to 1.23 mmol (CO:H_2_O = 3:104) (Fig. 4c, Supplementary Table S1). As shown in Fig. 4d, the distribution of hydrocarbons was sensitive to the amount of water. A high CO:H_2_O ratio resulted in a high CO:H_2_ ratio, favoring both chain propagation and olefin formation. Hence, the C_12+_^=^ selectivity among hydrocarbons with the highest CO:H_2_O ratio of 3:1 reached the highest value of 47.7% (Supplementary Table S1). Supplementary Figure S12 shows the detailed hydrocarbon selectivity with CO:H_2_O ratios of 3:1, 3:4, and 3:104. Hydrocarbon distribution *via* KES in water was similar to that *via* FTS in water, with methane as major product. Only a small amount of olefins was formed with an o/p ratio of 0.21. Notably, the highest C_12+_^=^ yield (1.79 mmol) was still achieved under the CO/H_2_O ratio of 3:2. Effect of water on the hydrocarbon distribution over a constant pressure of 2 MPa ulteriorly corroborated the cause of the abnormal pressurization effect on hydrocarbon production (Fig. 3b).

The amounts of Pt/Mo_2_N and Ru particles also influenced the rates for WGS and FTS (Supplementary Figs. S13–14). When we halved the amount of Pt/Mo_2_N, the total yield drastically dropped to less than half of that under optimal condition. The amounts of Ru did not significantly affect the FTS process. We speculated that a certain amount of hydrogen was necessary to trigger and drive FTS, probably due to the rapid diffusion of produced hydrogen out of PEG and thus the limited activation over Ru. With double amount of Pt/Mo_2_N, elevated yield of CO_2_ was observed. For a fixed reaction condition, merely increasing the rate of WGS could induce a mismatch of WGS and FTS, as we discussed above, while the yield of hydrocarbon exhibited a more sensitive dependence on Ru amounts than that at a low conversion.

The influence of the PEG molecular weight on the catalytic performance was explored (Supplementary Figs. S15, 16). Total yield was slightly higher in PEG-200 and severely restricted in PEG-600, due to the increasing viscosity with the increasing molecular weight. Though hydrocarbon yields, which were not as high as that in PEG-400, followed the trend discussed above, olefin-preference was still observed in PEG with different molecular weights.

Stability of this proposed system was also a necessity for practical application. However, Ru particles floating in viscous PEG phase during the extraction process led to the difficulty in separation of active components (Supplementary Fig. S17), and thus the stability test with multiple rounds. A *quasi*-stability test by studying the evolution of products for different reaction time became our alternative approach. With the prolonged reaction time, the yield of CO_2_ continuously increased at a diminishing rate due to the consumption of both CO and water, whereas the production of hydrocarbons steadily grew (Supplementary Fig. S18). The o/p ratio (for all C_2+_ products) increased from 1.00 for 2.5 h to 2.13 for 10 h (Supplementary Table S1). Chain propagation was also favored for longer reaction time, evidenced by the increasing selectivity for C_5+_ hydrocarbons over time (Supplementary Fig. S19). Detailed hydrocarbon selectivities for 2.5, 5, and 7.5 h are shown in Supplementary Fig. S20a–c. We ascribed the decreasing WGS rate to the consumption of CO and accumulation of CO_2_ in gas instead of rapid deactivation. Otherwise, the yield of CO_2_ should undergo a cliff drop. A restricted FTS rate was also observed at the initial stage, consistent with our observation of the results with half the amount of Pt/Mo_2_N (Supplementary Fig. S13). The XRD pattern and XPS spectra of the spent catalyst also suggested the structural stability of Mo_2_N and the metallic states of Pt and Ru (Supplementary Fig. S21). Hence, we speculated that stability during the reaction could be expected. The variation of C 1*s* spectrum was ascribed to that the formation of CH_*x*_ on surface might expel PEG during the washing procedure. The Ru 3*d*_5/2_ peak did not exhibit a measurable shift, compared with the fresh Pt/Mo_2_N-Ru sample with PEG residue, indicating PEG did not serve as an electronic promoter for Ru.

On account of the proof-of-concept nature of our strategy, we also extended our catalysts to non-noble metals to address the concern about the use of noble metals. It is generally accepted that Cu/ZnO/Al_2_O_3_ (CZA) serves as an efficient WGS catalyst^35,36^, while Co/Al_2_O_3_ is regarded as an active FTS catalyst. We physically mixed CZA with Co/Al_2_O_3_ for the KES reaction at 240 °C under 3 MPa of CO in comparison with standalone CZA or Co/Al_2_O_3_. CZA was only active towards WGS but inert towards FTS, resulting in the absence of hydrocarbons. As for Co/Al_2_O_3_, the WGS activity was rather poor (Supplementary Table S1). The insufficient hydrogen supply restrained the FTS process over Co/Al_2_O_3_, leading to low yields of hydrocarbons and especially C_12+_^=^ (Supplementary Table S1 and Figs. S23-24). Though the activity and C_12+_^=^ selectivity of CZA-Co/Al_2_O_3_ were lower than those of Pt/Mo_2_N-Ru, the great matching of WGS and FTS was also achieved with an o/p ratio of 4.19, indicating the universality of our strategy to tune selectivity of paraffins to olefins. Besides, iron-based catalysts themselves are both WGS active in the phase of iron oxides and FTS active in the phase of iron carbides. To this end, we conducted KES experiments on Fe-based catalysts at 240 °C, a temperature considered hard for olefin desorption from iron surface^37^. The olefin-preference was observed in spite of excessive CO_2_ production due to the strong WGS activity (Supplementary Table S1 and Fig. S25).

## Discussion

We showcased the potential of KES proceeded in a phase-transfer agent, PEG in our case, over Pt/Mo_2_N-Ru to produce extra-heavy olefins. KES provided continuously low hydrogen pressure, kinetically favoring the formation of olefins. PEG served as a selective extraction agent of olefins. Under an optimized condition, the kinetics of WGS and FTS were perfectly matched, as the yield ratio of CO_2_ to hydrocarbons reached the theoretical minimum of 2. Meanwhile, C_12+_^=^ yield reached its maximum of 1.79 mmol. Apart from Pt/Mo_2_N-Ru, we also extended to non-noble catalysts, proving the universality of this strategy. Compared with energy-intensive wax cracking process (>400 °C), severe operation conditions are not indispensable for this energy efficient KES route. KES route also enables the direct production of C_12+_^=^, circumventing the multi-step SHOP.

## Methods

### Chemicals and materials

(NH_4_)_6_Mo_7_O_24_·4H_2_O, H_2_PtCl_4_·6H_2_O, RuCl_3_·*x*H_2_O (Ru amount: ~37%), FeCl_3_·6H_2_O, Co(NO_3_)_2_·6H_2_O, cyclohexane, ethanol, ethylene glycol, polyethylene glycol-200 (PEG-200), polyethylene glycol-400 (PEG-400), polyethylene glycol-600 (PEG-600) were purchased from Sinopharm Chemical Reagent Co., Ltd. (Shanghai, China). 1-dodecene was purchased from Shanghai Aladdin Biochemical Technology Co., Ltd. (Shanghai, China). Al_2_O_3_ (catalyst support, high surface area, 1/8 pellets) was purchased from Alfa Aesar(China) Chemcals Co., Ltd. (Shanghai, China). NH_3_, N_2_, CO (with 5% N_2_ as the internal standard), CO_2_, H_2_, propylene, and syngas (H_2_:CO:Ar =64:32:4) were purchased from Nanjing Special Gas Factory Co., Ltd. (Nanjing, China). Syngas (H_2_:CO:N_2_ = 9.6:85.1:4.3) was compounded manually. Propylene-containing syngas (H_2_:CO:C_3_H_6_ = 14:14:2) was compounded manually. 50-mL Hastelloy slurry reactors were purchased from Shanghai Yanzheng Experiment Instrument Co., Ltd. (Shanghai, China).

### Synthesis of Mo_2_N

Typically, (NH_4_)_6_Mo_7_O_24_·4H_2_O was calcined at 500 °C for 6 h in a muffle furnace to obtain MoO_3_. MoO_3_ was then nitrided in 1 bar of pure NH_3_ flow with a flow rate of 50 mL min^−1^ in a tube furnace. The tube was heated with a heating ramp rate of 5 °C min^−1^ to 500 °C, and then a rate of 1 °C min^−1^ to 750 °C. After being maintained at 750 °C for 9 h, the sample was naturally cooled to room temperature, followed by passivation in 1 bar of CO_2_ flow with a flow rate of 50 mL min^−1^ for 1 h.

### Synthesis of Pt/Mo_2_N

500 mg of Mo_2_N was dispersed in 200 mL of ethanol. 40 mL of H_2_PtCl_4_·6H_2_O aqueous solution (0.00128 mol L^−1^) and 40 mL of NaBH_4_ ethanol solution (0.0102 mol L^−1^) were added dropwise with stirring into Mo_2_N suspension simultaneously with a rate of 20 mL h^−1^ through a two-channel syringe pump at room temperature. The as-synthesized sample was washed with ethanol thrice to remove the residual ions, followed by drying at 60 °C under vacuum.

### Synthesis of Pt/Mo_2_N-Ru

Typically, after 100 mg of Pt/Mo_2_N was dispersed in 15 mL of PEG-400/water binary solvent (with 142, 284, and 426 μL of water for CO:H_2_O ratio of 3:1, 3:2, and 3:4 respectively) in a 50-mL Hastelloy slurry reactor, 100 mg of RuCl_3_·*x*H_2_O was added with stirring. After the reactor was pressurized with H_2_ (1.0 MPa) at room temperature, the reactor was heated to 150 °C, then kept for 5 h, and cooled down to room temperature. Samples with other ratios of Pt/Mo_2_N to Ru were synthesized similarly.

### Synthesis of Ru particles

100 mg of RuCl_3_·*x*H_2_O was added into 15 mL of PEG-400 in a 50-mL Hastelloy slurry reactor with stirring. After the reactor was pressurized with H_2_ (1.0 MPa) at room temperature, the reactor was heated to 150 °C, then kept for 5 h, and cooled down to room temperature. For FTS conducted in water, the as-synthesized particles were washed with water thrice to remove the residual PEG, and then transferred into a reactor with 15 mL of water.

### Synthesis of Ru/Pt/Mo_2_N

200 mg of Pt/Mo_2_N was dispersed in 200 mL of ethanol. 50 mL of RuCl_3_·*x*H_2_O aqueous solution (4 mg mL^−1^) and 50 mL of NaBH_4_ ethanol solution (0.128 mol L^−1^) were added dropwise with stirring into Pt/Mo_2_N suspension simultaneously with a rate of 25 mL h^−1^ through a two-channel syringe pump at room temperature. The as-synthesized sample was washed with ethanol thrice to remove the residual ions, followed by drying at 60 °C under vacuum.

### Synthesis of Fe-based catalyst

10 mL of 2.4 M KBH_4_ aqueous solution was added dropwise to 30 mL of 0.2 M FeCl_3_·6H_2_O ethylene glycol solution with a rate of 20 mL h^−1^ through a syringe pump under an inert atmosphere of N_2_. A magnet was used to separate Fe precipitate from solution. The precipitate was washed by ethanol 8 times to avoid the oxidation and remove the possible K residue.

### Synthesis of Co/Al_2_O_3_

Co/Al_2_O_3_ was prepared by impregnating Co(NO_3_)_2_·6H_2_O onto Al_2_O_3_ powder, followed by hydrogen reduction at 600 °C for 5 h.

### Extraction of 1-dodecene

95 mg of 1-dodecene was dispersed into a centrifuge tube containing 15 mL of PEG-400. 2 mL of cyclohexane was then added at room temperature, followed by vigorous shaking for 1 min. For the water-aided extraction, 10 mL of water was added into the mixture of cyclohexane and PEG at room temperature, followed by vigorous shaking for 1 min. 1-dodecene dissolved in cyclohexane was determined by gas chromatograph (Shimadzu GC-2014).

### Catalytic tests

All FTS and KES reactions were carried out in a 50-mL Hastelloy slurry reactor.

For FTS conducted in PEG-400, after the reduction of RuCl_3_·*x*H_2_O, the reactor was washed with N_2_ for 5 times to remove H_2_ residual and then pressurized with 2 MPa of syngas (H_2_/CO = 2/1 or 1/9) at room temperature. For FTS conducted in water, after the Ru particles were washed with water and transferred into a reactor, the reactor was washed with N_2_ for 5 times to remove air residual and then pressurized with 2 MPa of syngas (H_2_/CO = 2/1) at room temperature. For propylene hydroformaylation conducted in PEG-400, after the reduction of RuCl_3_·*x*H_2_O, the reactor was washed with N_2_ for 5 times to remove H_2_ residual and then pressurized with 3 MPa of propylene-containing syngas (H_2_/CO/C_3_H_6_ = 14/14/2) at room temperature.

For typical KES test over Pt/Mo_2_N-Ru, the reactor was washed with N_2_ for 5 times after the synthesis of Pt/Mo_2_N-Ru (typically, 100 mg of Pt/Mo_2_N and ~37 mg of Ru particles) or the addition of 137 mg of Ru/Pt/Mo_2_N to remove hydrogen or air residual. Then the reactor was pressurized with 2, 3, or 4 MPa of CO at room temperature. All solvents (pure water, or PEG/water binary solvent) were kept to 15 mL. 284, 426, and 568 μL of water were used for 2, 3, and 4 MPa of CO while CO:H_2_O ratio was kept to 3:2. 142, 284, 568, and 15,000 μL of water were used for CO:H_2_O ratio of 3:1, 3:2, 3:4, and 3:104 while the pressure of CO was kept to 2 MPa. Procedure of KES tests over other catalysts was similar.

For one specified catalytic condition, two reactors were used. For accurate quantitation of total CO_2_ (gaseous CO_2_ and dissolved CO_2_), one reactor was heated and connected to a sealed container with 20 mL of NaOH aqueous solution (3.0 mol L^−1^). After CO_2_ was entirely absorbed, 50 mL of Ba(NO_3_)_2_ aqueous solution (0.5 mol L^−1^) was added. The precipitated BaCO_3_ was washed thrice and dried at 80 °C overnight. The dried sample was weighed to calculate the amount of formed CO_2_. For quantitation of other gaseous compositions (CO and gaseous C_1-7_ hydrocarbons), the other reactor was connected to a gas chromatograph (Shimadzu GC-2014). CO and CH_4_ were analyzed by using a carbon molecular sieve column (TDX-1) with a thermal conductivity detector (TCD). The conversion of CO was determined by the internal standard merely for reference since dissolution in PEG might obscure the quantitation, while the procedure of accurate quantitation of hydrocarbons was as follow. Gaseous hydrocarbons were analyzed using an Al_2_O_3_ capillary column with a flame ionization detector (FID) and a PONA capillary column with an FID. After quantitation of gaseous products, a certain amount of cyclohexane (typically 2 mL) was added into the reactor without stirring. After carefully transferring the liquid components (cyclohexane and PEG phases) and solid components (WGS catalysts and FTS catalysts) into a centrifuge tube, 10 ml of water was then added followed by 1-min shaking. The lid of centrifuge tube should be pressed hard during shaking, to prevent the dissolved CO_2_ to pop out of the centrifuge tube and cause the loss of products. Cyclohexane solution was injected into a vaporization chamber in GC and then analyzed using a PONA capillary column with an FID. CH_4_ was taken as a reference bridge between TCD and FID.

Hydrocarbons were calculated on a carbon-atom basis.

The selectivity for hydrocarbon C_n_H_m_ was obtained according to

4 $${{{{{{\rm{C}}}}}}}_{{{{{{\rm{n}}}}}}}{{{{{{\rm{H}}}}}}}_{{{{{\rm{{m}}}}}}}\,{{{{{\rm{selectivity}}}}}}=\frac{{{{{{\rm{n}}}}}}{{{{{\rm{C}}}}}}_{{{{{\rm{n}}}}}}{{{{{\rm{H}}}}}}_{{{{{\rm{m}}}}}}}{\mathop{\sum }\limits_{i}{{{{{\rm{iC}}}}}}_{{{{{\rm{i}}}}}}{{{{{\rm{H}}}}}}_{{{{{\rm{m}}}}}}}\times 100\%$$

In Eq. (4), C_n_H_m_ represents moles of individual hydrocarbon products. Other possible oxygenate products were under the detection limit. The carbon balance was over 95%.

### Characterizations

XRD patterns were recorded by using a Philips X’Pert Pro Super diffractometer with Cu-Kα radiation (λ = 1.54178 Å). XPS measurements were conducted on an ESCALAB 250 (Thermo-VG Scientific, USA) with an Al Kα X-ray source (1486.6 eV protons) in Constant Analyser Energy (CAE) mode with a pass energy of 30 eV for all spectra. ICP-AES (Atomscan Advantage, Thermo Jarrell Ash, USA) was used to determine the loading amount of Pt. TEM images were taken using a Hitachi H-7700 transmission electron microscope at an acceleration voltage of 100 kV. HAADF analysis was collected on a Titan Themis Z transmission electron microscope with double aberration.

## Supplementary information

Supplementary information

## Data Availability

The data that support the findings of this study are available from the corresponding author upon reasonable request. Source data are provided with this paper.

## Source data

Source Data
