# Supplementary material for: Direct synthesis of extra-heavy olefins from carbon monoxide and water
Source: Nat Commun. 2023 Apr 3;14:1857. doi: 10.1038/s41467-023-37599-2 (PMC10070633; doi:10.1038/s41467-023-37599-2)
Supplement: Supplementary file 1 — Supplementary information [file 41467_2023_37599_MOESM1_ESM.pdf]

Supplementary Information for

Direct Synthesis of Extra-heavy Olefins from Carbon Monoxide and Water

Chuanhao Wang[1†], Junjie Du[1†], Lin Zeng[1], Zhongling Li[1], Yizhou Dai[1], Xu Li[1], Zijun Peng[1], Wenlong Wu[1], Hongliang Li[1*], Jie Zeng[1,2*]

[1]Hefei National Research Center for Physical Sciences at the Microscale, Key Laboratory of Strongly-Coupled Quantum Matter Physics of Chinese Academy of Sciences, Key Laboratory of Surface and Interface Chemistry and Energy Catalysis of Anhui Higher Education Institutes, Department of Chemical Physics, University of Science and Technology of China, Hefei, Anhui 230026, P. R. China

[2]School of Chemistry & Chemical Engineering, Anhui University of Technology, Ma'anshan, Anhui 243002, P. R. China

[†]These authors contributed equally: Chuanhao Wang, Junjie Du.

[*]e-mail: lihl@ustc.edu.cn; zengj@ustc.edu.cn.

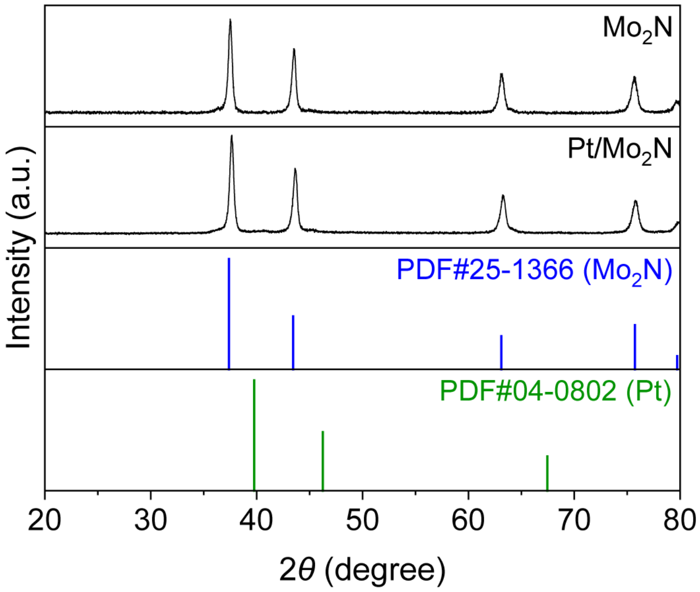

Supplementary Figure S1 | XRD patterns of Mo_2N and Pt/Mo_2N.

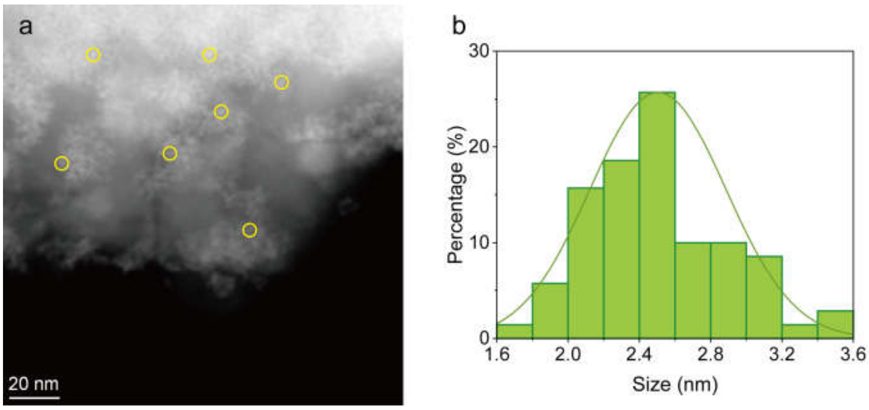

Supplementary Figure S2 | Size distribution of Pt particles supported on Mo_2N. (a) HAADF-STEM images of Pt/Mo_2N. (**b**) Size distribution of Pt particles.

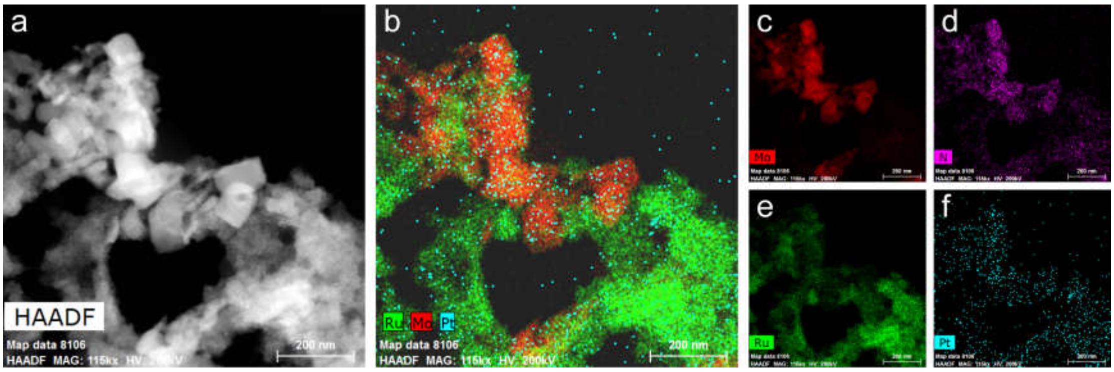

Supplementary Figure S3 | STEM-EDX elemental mapping images of Pt/Mo_2N-Ru. (a) HAADF-STEM image. (b-f) STEM-EDX elemental mapping image. Red represents Mo. Purple represents N. Green represents Ru. Blue represents Pt.

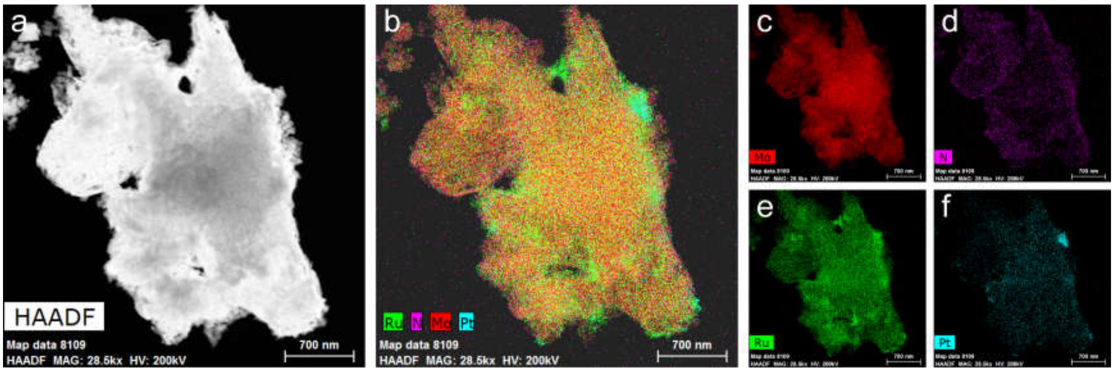

Supplementary Figure S4 | STEM-EDX elemental mapping images of Ru/Pt/Mo_2N. (a) HAADF-STEM image. (b-f) STEM-EDX elemental mapping image. Red represents Mo. Purple represents N. Green represents Ru. Blue represents Pt. Red represents Mo. Purple represents N. Green represents Ru. Blue represents Pt.

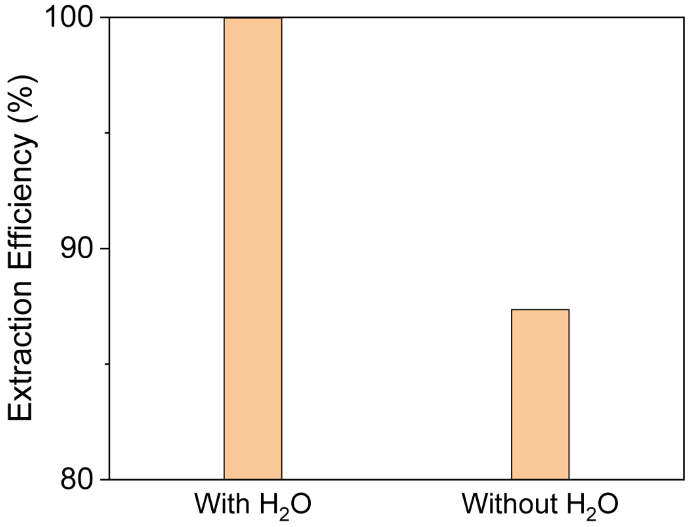

Supplementary Figure S5 | Extraction of 1-dodecene using cyclohexane with or without the aid of water. 95 mg of 1-dodecene was dispersed into 15 mL of PEG. 2 mL of cyclohexane (with or without 10 mL of water) was used to extract 1-dodecene from PEG.

Supplementary Table S1 | Catalytic performance towards KES.

Entry	Catalyst	Reaction conditions					Catalytic properties						
		Solvent	CO pressure (MPa)	CO/H_2O	Temperature (°C)	Reaction time (h)	Conversion (%)	CO_2 yield (mmol)	HC yield[a] (mmol)	CO_2/HC	o/p ratio (C_{2+})	$C_{12+}^{=}$ selectivity (%)	$C_{12+}^{=}$ yield (mmol)
1	Pt/Mo_2N-Ru	PEG-400	2	3:2	200	10	52.55	8.63	4.42	1.95	2.13	40.4	1.79
2	Pt/Mo_2N-Ru	PEG-400	3	3:2	200	10	40.28	10.61	3.43	3.09	2.17	41.9	1.44
3	Pt/Mo_2N-Ru	PEG-400	4	3:2	200	10	76.60	30.46	5.81	5.24	1.30	23.2	1.35
4	Ru/Pt/Mo_2N	PEG-400	2	3:2	200	10	61.72	13.70	0.27	50.74	0.83	17.9	0.05
5	Pt/Mo2N-Ru	PEG-400	2	3:2	200	2.5	18.08	3.67	0.90	4.08	1.00	28.7	0.26
6	Pt/Mo_2N-Ru	PEG-400	2	3:2	200	5	35.97	6.78	1.97	3.44	1.47	38.5	0.76
7	Pt/Mo_2N-Ru	PEG-400	2	3:2	200	7.5	45.97	7.99	3.37	2.37	2.23	39.5	1.33
8	Pt/Mo_2N-Ru	PEG-400	2	3:1	200	10	44.95	7.18	2.36	3.04	2.38	47.7	1.13
9	Pt/Mo_2N-Ru	PEG-400	2	3:4	200	10	75.68	13.19	4.46	2.96	2.17	38.6	1.72
10	Pt/Mo_2N-Ru	H_2O	2	3:104	200	10	80.47	17.22	1.23	13.96	0.21	5.65	0.07
11	50%Pt/Mo_2N-50%Ru[c]	PEG-400	2	3:2	200	10	16.78	2.99	0.42	7.05	2.95	23.21	0.10
12	50%Pt/Mo_2N-200%Ru[c]	PEG-400	2	3:2	200	10	18.02	3.05	0.50	6.08	2.56	25.8	0.13
13	200%Pt/Mo_2N-50%Ru[c]	PEG-400	2	3:2	200	10	94.85	19.54	2.65	7.38	2.44	17.7	0.47
14	200%Pt/Mo_2N-200%Ru[c]	PEG-400	2	3:2	200	10	97.51	19.77	3.25	6.09	2.88	28.08	0.91
15	Pt/Mo_2N-Ru	PEG-200	2	3:2	200	10	64.52	13.29	2.35	5.66	3.09	33.9	0.80
16	Pt/Mo_2N-Ru	PEG-600	2	3:2	200	10	21.89	4.12	0.58	7.15	3.17	28.3	0.16
17	CZA-Co/Al_2O_3	PEG-400	3	3:2	240	10	24.51	4.78	2.33	2.05	4.19	21.9	0.51
18	Co/Al_2O_3	PEG-400	3	3:2	240	10	7.24	1.03	0.056	18.35	1.10	0.64	0.00036
19	CZA[b]	PEG-400	3	3:2	240	10	19.86	6.71	N.A.	-	-	-	-
20	Fe-based catalyst	PEG-400	3	3:2	240	10	68.95	20.84	2.14	9.75	3.64	7.48	0.16

[a]HC represents hydrocarbons.

[b]CZA represents commercial $Cu/ZnO/Al_2O_3$.

[c]The amount of Pt/Mo_2N and Ru can be found in **Supplementary Figure S13-14**.

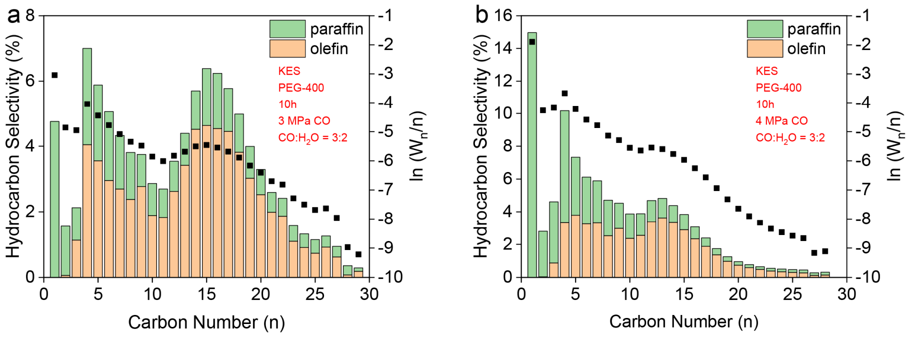

Supplementary Figure S6 | Detailed hydrocarbon selectivity of KES under different pressure of CO. (**a**) KES over Pt/Mo_2N-Ru under 3 MPa of CO. (**b**) KES over Pt/Mo_2N-Ru under 4 MPa of CO. All catalytic experiments were conducted in a 50-mL Hastelloy slurry reactor at 200 °C for 10 h with a CO:H_2O ratio of 3:2. Total volume of PEG solvent (with 426 and 568 μL of water for 3 and 4 MPa of CO) was kept to 15 mL. The used Pt/Mo_2N-Ru catalyst contained 100 mg of Pt/Mo2N and ~37 mg of Ru particles.

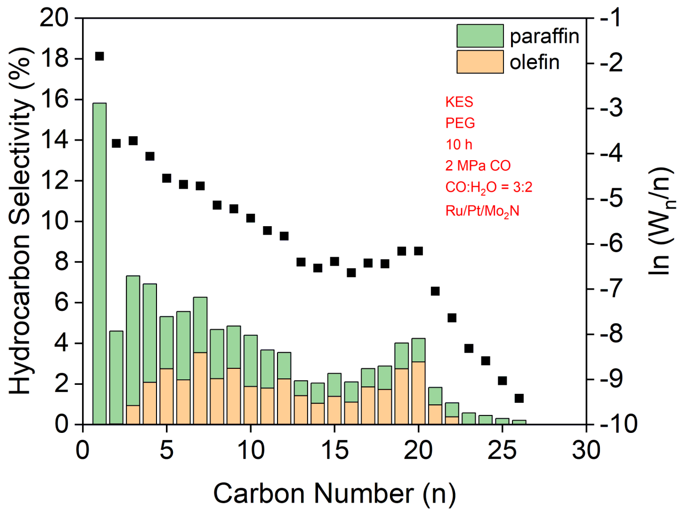

Supplementary Figure S7 | Detailed hydrocarbon selectivity of KES over Ru/Pt/Mo_2N under 2 MPa of CO. Catalytic experiment was conducted in a 50-mL Hastelloy slurry reactor under 2 MPa of CO with a CO:H_2O ratio of 3:2 at 200 °C for 10 h. Total volume of PEG solvent (with 284 µL of water) was kept to 15 mL. 137 mg of Ru/Pt/Mo_2N was used.

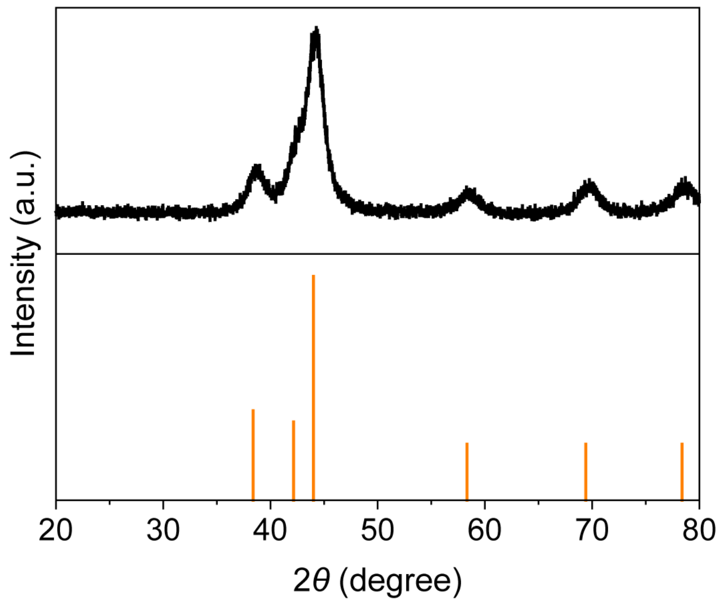

Supplementary Figure S8 | XRD pattern of Ru particles.

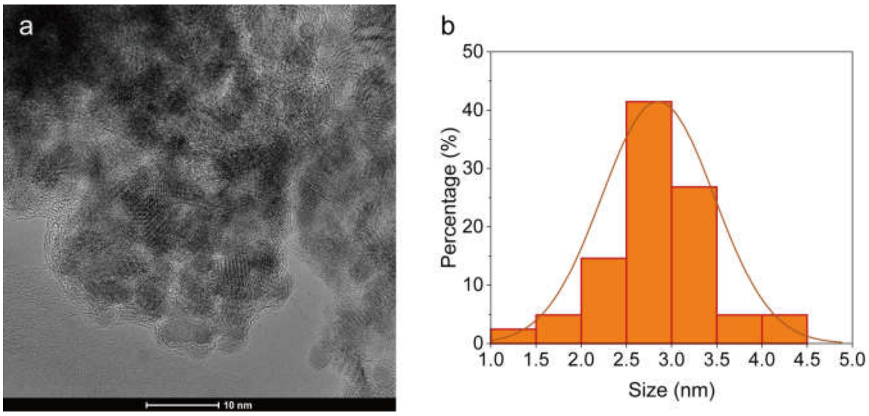

Supplementary Figure S9 | Size distribution of Ru particles. (**a**) HRTEM images of Ru particles. (**b**) Size distribution of Ru particles.

Supplementary Table S2 | Catalytic performances of FTS and hydroformylation.

Entry	Catalyst	Reaction conditions					Catalytic properties				
		Solvent	Pressure (MPa)	CO/H_2	Temperature (ºC)	Reaction time (h)	Conversion (%)	CO_2 yield (mmol)	HC yield (mmol)	o/p ratio (C_{2+})	$C_{12+}^{=}$ selectivity (%)
1	Ru particles	PEG	2	9:1	200	10	20.68	1.09	2.14	2.17	35.9
2	Ru particles	PEG	2	1:2	200	10	88.47	0.62	6.39	0.92	14.9
3	Ru particles	H_2O	2	1:2	200	10	95.92	0.63	7.06	0.01	0.3
4	Ru particles	PEG	3	1:1[a]	200	10	42.48	2.00	4.84	1.45	8.74

Oxygenates were under the detection limit of GC.

[a]Hydroformylation condition: $CO/H_2/C_3H_6$ = 14:14:2.

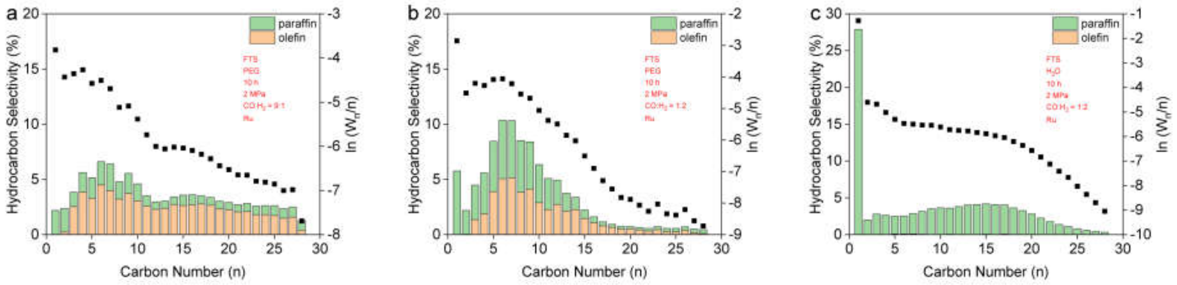

Supplementary Figure S10 | Detailed hydrocarbon selectivity of FTS over Ru particles under 2 MPa of syngas. (**a**) FTS in PEG under 2 MPa of syngas (CO/H_2 = 9:1). (**b**) FTS in PEG under 2 MPa of syngas (CO/H_2 = 1:2). (**c**) FTS in water under 2 MPa of syngas (CO/H_2 = 1:2). All catalytic experiments were conducted in a 50-mL Hastelloy slurry reactor at 200 °C for 10 h. Total volume of solvent (PEG or water) was kept to 15 mL. 37mg of Ru particles were used as catalysts.

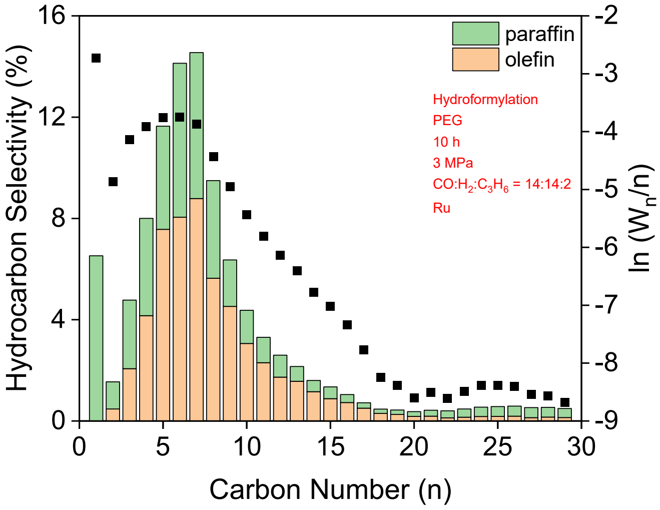

Supplementary Figure S11 | Detailed hydrocarbon selectivity of hydroformylation over Ru particles. Hydroformylation was conducted in a 50-mL Hastelloy slurry reactor at 200 °C for 10 h under 3 MPa of propylene-containing syngas ($CO/H_2/C_3H_6$ = 14:14:2). Volume of PEG was kept to 15 mL. 37mg of Ru particles were used as catalysts. Due to the excessive signal of unreacted propylene, yield and o/p ratio of C_3 species were estimated by averaging those of C_2 and C_4 species.

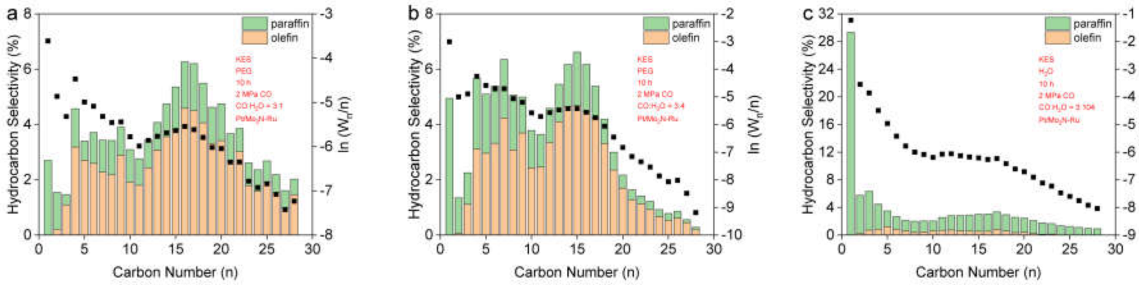

Supplementary Figure S12 | Detailed hydrocarbon selectivity of KES for different CO/H_2O ratios. (**a**) CO/H_2O = 3/1. (**b**) CO/H_2O = 3/4. (**c**) CO/H_2O = 3/104. All catalytic experiments were conducted in a 50-mL Hastelloy slurry reactor under 2 MPa of CO at 200 °C for 10 h. Total volume of solvents (a, PEG with 142 µL of water; b, PEG with 568 µL of water; c, pure water) was kept to 15 mL. The used Pt/Mo_2N-Ru catalyst contained 100 mg of Pt/Mo_2N and ~37mg of Ru particles.

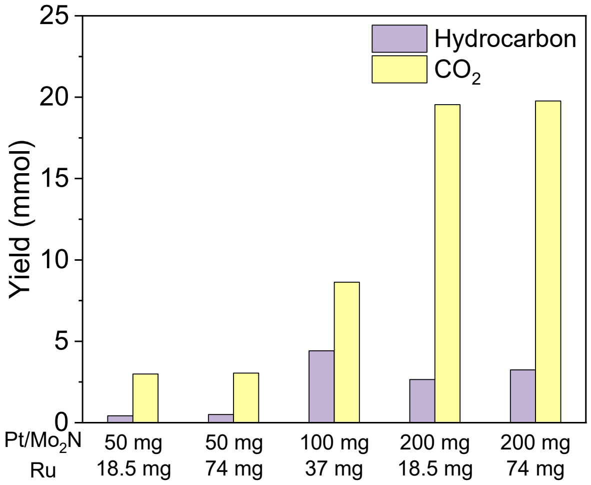

Supplementary Figure S13 | Yields of hydrocarbons and CO_2 of KES over Pt/Mo_2N-Ru with different amounts of Pt/Mo_2N and Ru. All catalytic experiments were conducted in a 50-mL Hastelloy slurry reactor at 200 °C under 2 MPa of CO with a CO:H_2O ratio of 3:2 for 10 h. Total volume of PEG solvent, with 284 µL of water, was kept to 15 mL.

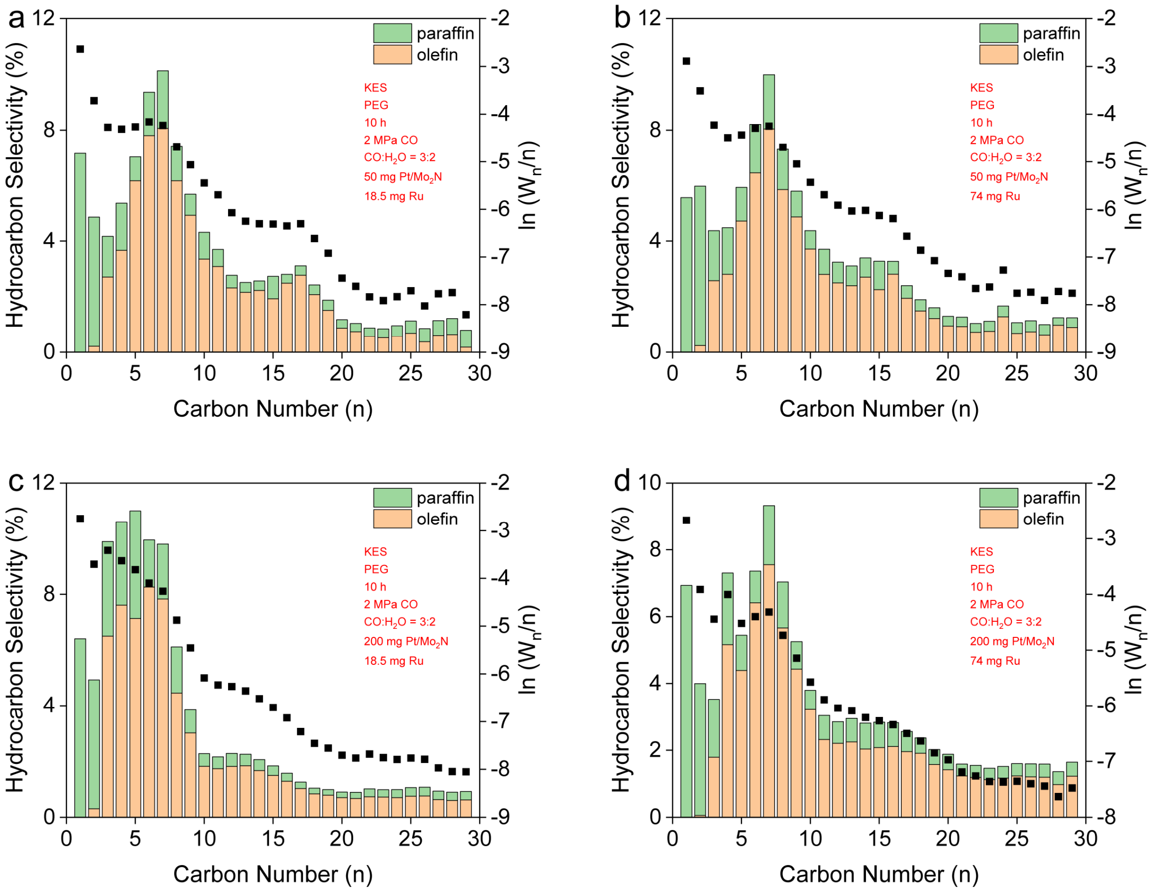

Supplementary Figure S14 | Detailed hydrocarbon selectivity of KES over Pt/Mo_2N-Ru with different amounts of Pt/Mo_2N and Ru. (**a**) 50 mg Pt/Mo_2N and 18.5 mg Ru. (**b**) 50 mg Pt/Mo_2N and 74 mg Ru. (**c**) 200 mg Pt/Mo_2N and 18.5 mg Ru. (**d**) 200 mg Pt/Mo_2N and 74 mg Ru. All catalytic experiments were conducted in a 50-mL Hastelloy slurry reactor at 200 °C under 2 MPa of CO with a CO:H_2O ratio of 3:2 for 10 h. Total volume of PEG solvent, with 284 μL of water, was kept to 15 mL. The used Pt/Mo_2N-Ru catalyst contained 100 mg of Pt/Mo_2N and ~37 mg of Ru particles.

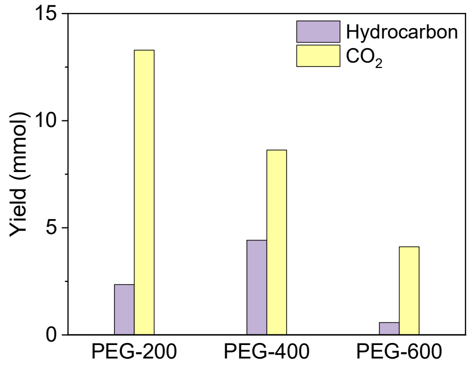

Supplementary Figure S15 | Yields of hydrocarbons and CO_2 of KES over Pt/Mo_2N-Ru in PEG with different molecular weights. All catalytic experiments were conducted in a 50-mL Hastelloy slurry reactor at 200 °C under 2 MPa of CO with a CO:H_2O ratio of 3:2 for 10 h. Total volume of PEG solvent, with 284 μL of water, was kept to 15 mL. The used Pt/Mo_2N-Ru catalyst contained 100 mg of Pt/Mo_2N and ~37mg of Ru particles.

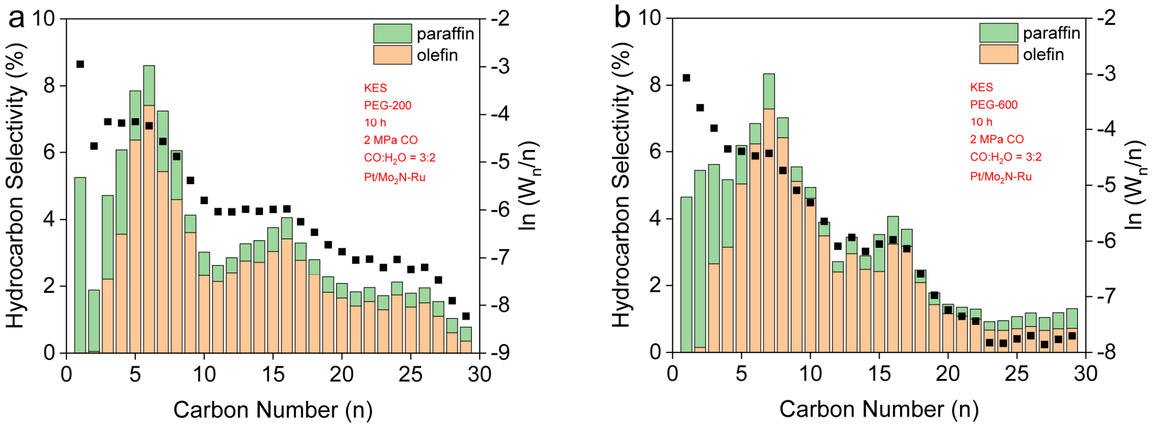

Supplementary Figure S16 | Detailed hydrocarbon selectivity of KES over Pt/Mo_2N-Ru in PEG with different molecular weights. (**a**) PEG-200. (**b**) PEG-600. All catalytic experiments were conducted in a 50-mL Hastelloy slurry reactor at 200 °C under 2 MPa of CO with a CO:H_2O ratio of 3:2 for 10 h. Total volume of PEG solvent, with 284 μL of water, was kept to 15 mL. The used Pt/Mo_2N-Ru catalyst contained 100 mg of Pt/Mo_2N and ~37 mg of Ru particles.

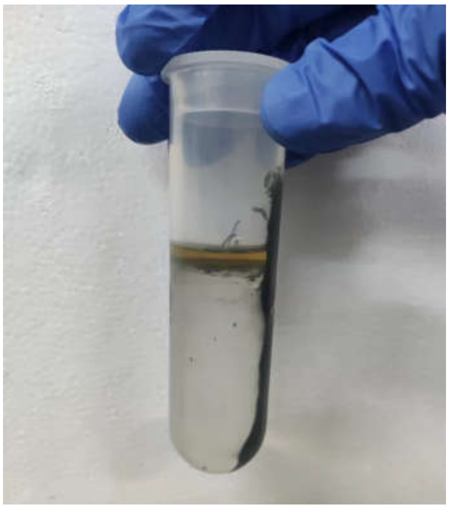

Supplementary Figure S17 | Extraction of hydrocarbon products from PEG to cyclohexane with the aid of water. After water had been added into the mixture of PEG, cyclohexane, and the solid catalyst, centrifugation was conducted to accelerate extraction and separate solid catalyst from cyclohexane phase. Though some Ru particles still suspended in the viscous PEG, close to the interface of PEG and cyclohexane.

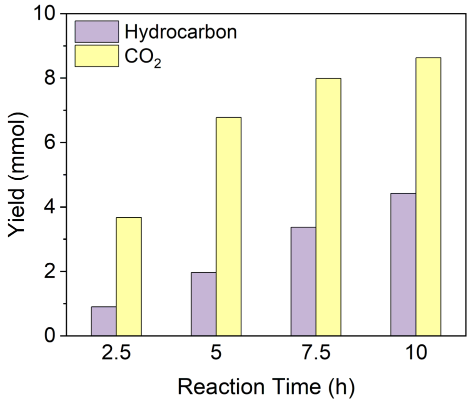

Supplementary Figure S18 | Yields of hydrocarbons and CO_2 of KES over Pt/Mo_2N-Ru with different reaction time. From left to right: 2.5, 5, 7.5, and 10 h. All catalytic experiments were conducted in a 50-mL Hastelloy slurry reactor at 200 °C under 2 MPa of CO with a CO:H_2O ratio of 3:2. Total volume of PEG solvent, with 284 μL of water, was kept to 15 mL. The used Pt/Mo_2N-Ru catalyst contained 100 mg of Pt/Mo_2N and ~37 mg of Ru particles.

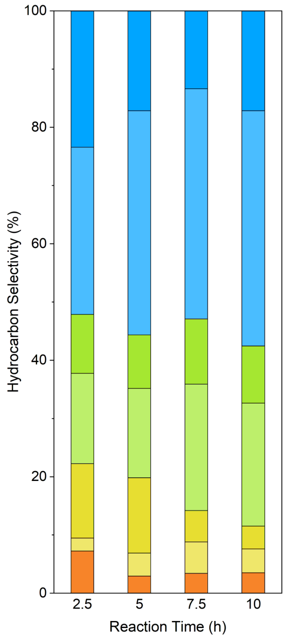

Supplementary Figure S19 | Fractional hydrocarbon selectivity of KES over Pt/Mo_2N-Ru with different reaction time. From left to right: 2.5, 5, 7.5, and 10 h. All catalytic experiments were conducted in a 50-mL Hastelloy slurry reactor at 200 °C under 2 MPa of CO with a CO:H_2O ratio of 3:2. Total volume of PEG solvent, with 284 µL of water, was kept to 15 mL. The used Pt/Mo_2N-Ru catalyst contained 100 mg of Pt/Mo_2N and ~37 mg of Ru particles. Colors represent the same products as those in Figure 3b.

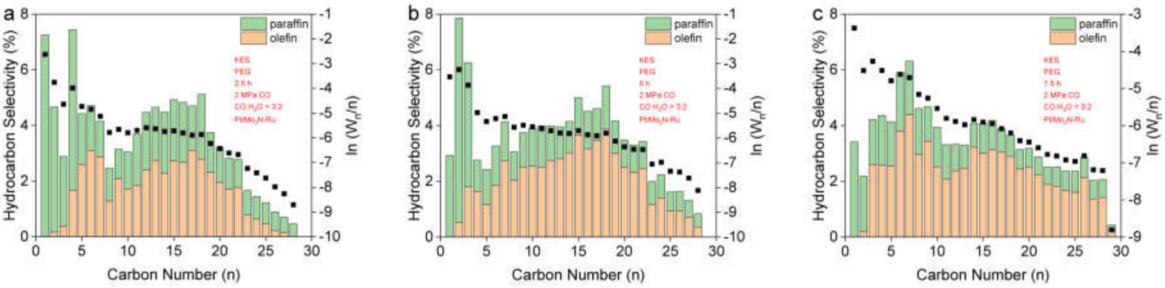

Supplementary Figure S20 | Detailed hydrocarbon selectivity of KES over Pt/Mo_2N-Ru with different reaction time. Detailed hydrocarbon selectivity of KES over Pt/Mo_2N-Ru after (**a**) 2.5 h, (**b**) 5 h, and (**c**) 7.5 h. All catalytic experiments were conducted in a 50-mL Hastelloy slurry reactor at 200 °C under 2 MPa of CO with a CO:H_2O ratio of 3:2. Total volume of PEG solvent, with 284 μL of water, was kept to 15 mL. The used Pt/Mo_2N-Ru catalyst contained 100 mg of Pt/Mo_2N and ~37 mg of Ru particles.

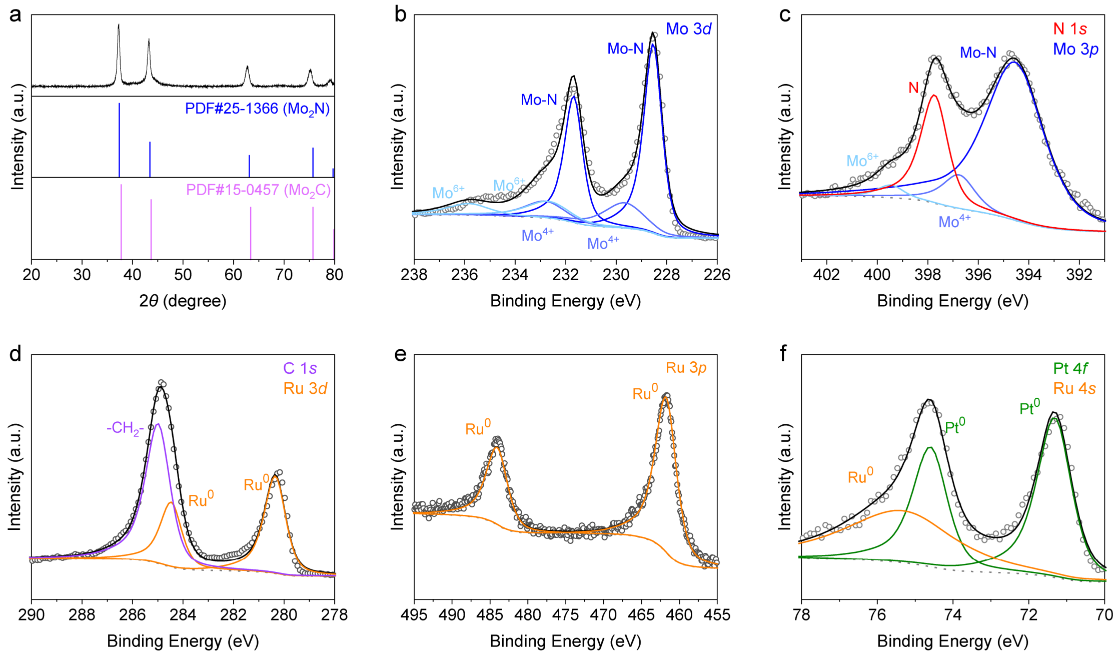

Supplementary Figure S21 | Structural characterizations of spent Pt/Mo_2N-Ru. (**a**) XRD pattern of spent Pt/Mo_2N-Ru. (**b**) Mo 3*d*, (**c**) N 1*s* and Mo 3*p*, (**d**) C 1*s* and Ru 3*d*, (**e**) Ru 3*p*, and (**f**) Pt 4*f* and Ru 4*s* XPS spectra of spent Pt/Mo_2N-Ru.

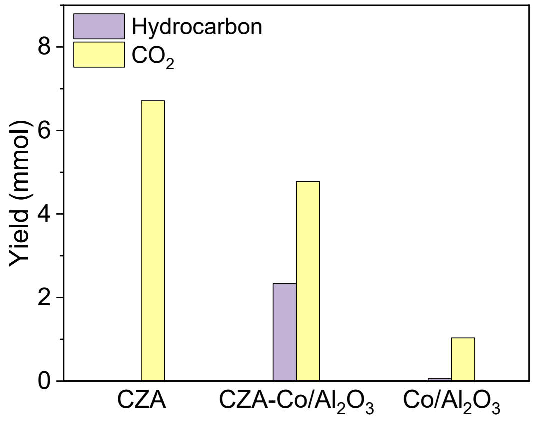

Supplementary Figure S22 | Yields of hydrocarbons and CO_2 of KES over CZA, CZA-Co/Al_2O_3, and Co/Al_2O_3. All catalytic experiments were conducted in a 50-mL Hastelloy slurry reactor at 240 °C under 3 MPa of CO with a CO:H_2O ratio of 3:2 for 10 h. Total volume of PEG solvent, with 426 μL of water, was kept to 15 mL.

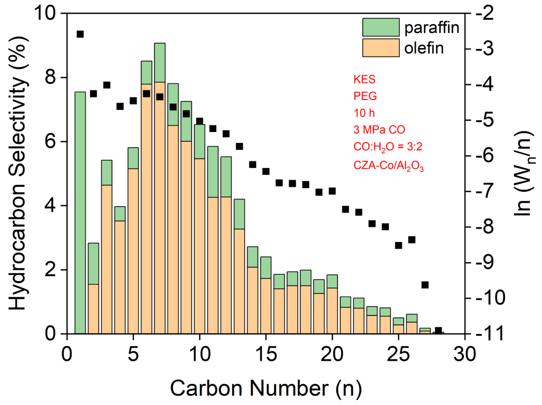

Supplementary Figure S23 | Detailed hydrocarbon selectivity of KES over CZA-Co/Al_2O_3. Catalytic experiments were conducted in a 50-mL Hastelloy slurry reactor at 240 °C under 3 MPa of CO with a CO:H_2O ratio of 3:2 for 10 h. Total volume of PEG solvent, with 426 μL of water, was kept to 15 mL. The used CZA-Co/Al_2O_3 catalyst contained 10 mg of CZA and 100 mg of Co/Al_2O_3 with Co loading of ~30%.

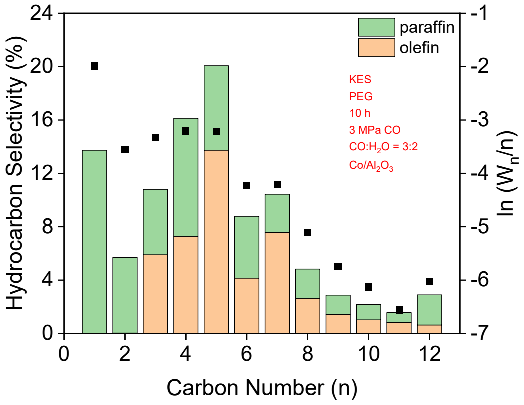

Supplementary Figure S24 | Detailed hydrocarbon selectivity of KES over Co/Al_2O_3. Catalytic experiments were conducted in a 50-mL Hastelloy slurry reactor at 240 °C under 3 MPa of CO with a $CO:H_2O$ ratio of 3:2 for 10 h. Total volume of PEG solvent, with 426 μL of water, was kept to 15 mL. 100 mg of Co/Al_2O_3 with Co loading of ~30% was used as the catalyst.

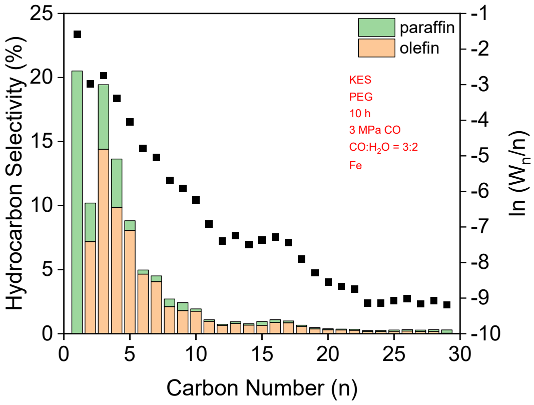

Supplementary Figure S25 | Detailed hydrocarbon selectivity of KES over Fe-based catalysts. Catalytic experiments were conducted in a 50-mL Hastelloy slurry reactor at 240 °C under 3 MPa of CO with a CO:H_2O ratio of 3:2 for 10 h. Total volume of PEG solvent, with 426 μL of water, was kept to 15 mL. 100 mg of Fe-based catalyst was used.
